# Peroxisome deficient invertebrate and vertebrate animal models

**DOI:** 10.3389/fphys.2013.00335

**Published:** 2013-11-22

**Authors:** Paul P. Van Veldhoven, Myriam Baes

**Affiliations:** ^1^LIPIT, Department Cellular and Molecular Medicine, KU LeuvenLeuven, Belgium; ^2^Laboratory of Cellular Metabolism, Department of Pharmaceutical and Pharmacological Sciences, KU LeuvenLeuven, Belgium

**Keywords:** inflammation, male fertility, phytanic acid, plasmalogens, PUFA, very long chain fatty acids, Zellweger syndrome

## Abstract

Although peroxisomes are ubiquitous organelles in all animal species, their importance for the functioning of tissues and organs remains largely unresolved. Because peroxins are essential for the biogenesis of peroxisomes, an obvious approach to investigate their physiological role is to inactivate a *Pex* gene or to suppress its translation. This has been performed in mice but also in more primitive organisms including *D. melanogaster*, *C. elegans*, and *D. rerio*, and the major findings and abnormalities in these models will be highlighted. Although peroxisomes are generally not essential for embryonic development and organogenesis, a generalized inactivity of peroxisomes affects lifespan and posthatching/postnatal growth, proving that peroxisomal metabolism is necessary for the normal maturation of these organisms. Strikingly, despite the wide variety of model organisms, corresponding tissues are affected including the central nervous system and the testis. By inactivating peroxisomes in a cell type selective way in the brain of mice, it was also demonstrated that peroxisomes are necessary to prevent neurodegeneration. As these peroxisome deficient model organisms recapitulate pathologies of patients affected with peroxisomal diseases, their further analysis will contribute to the elucidation of still elusive pathogenic mechanisms.

## Introduction

Absence of peroxisomes in man leads to a devastating disease, clinically known as the hepato-renal syndrome of Zellweger. Affected baby's are born alive, but are severely hypotonic, mentally retarded with brain malformation, liver and kidney problems, and die generally with the first weeks of life (Wanders and Waterham, 2005). Understanding the anomalies at the cellular and organ level and the malformation during development in such patients with a peroxisome biogenesis disorder, requires access to suitable experimental material. Unfortunately, for man the sources are rather limited (fibroblasts, lymphoblasts, amniotic villi), and not representative for specialized cells/tissues. In addition, no natural occurring or inducible animal model is known. Hence, as soon as appropriate molecular techniques were established, animal models were created, starting of with PEX5^1^ (Baes et al., 1997) and PEX2 (Faust and Hatten, 1997) deficient mice in 1997, followed later by inactivation of peroxins in other laboratory “pet-animals” like worms, fruitfly, or zebrafish.

In addition to these animal models, peroxisome deficient mutants were created in different yeasts, starting of with baker's yeast (Erdmann et al., 1989), followed by *Hansenula polymorpha* (Cregg et al., 1990) and *Pichia pastoris* (Gould et al., 1992); in filamentous fungi, *Neurospora crassa* (Sichting et al., 2003; Managadze et al., 2007), *Magnaporthe grisea* (Ramos-Pamplona and Naqvi, 2006), *Aspergillus oryzae* (Escano et al., 2009), in plants like *Arabidopsis* (Kaplan et al., 2001; Schumann et al., 2003; Fan et al., 2005), in trypanosomes (Banerjee et al., 2005; Galland et al., 2007). Some of the latter models are described elsewhere in this book, whereas for a treatise on human disorders linked to peroxisomes we refer to (Wanders and Waterham, 2005; Waterham and Ebberink, 2012).

Before discussing in more detail the different animal models, a general description of the metabolic functions of peroxisomes is given, followed by a short note about their biogenesis.

### Peroxisomal metabolism

From a human pathological point of view, the main peroxisomal pathways are β-oxidation, α-oxidation, and ether lipid synthesis, and to a lesser extent glyoxylate metabolism and xanthine metabolism. Whereas peroxisomal β-oxidation seems universally present in all animals, although sometimes serving other purposes, some of the other pathways might be missing in lower vertebrates/invertebrates (e.g., etherlipid synthesis). In the following paragraphs the main pathways are briefly described, whereas their specific roles, if known, will be highlighted when discussing the different models (enzymes are named according to the mouse nomenclature).

**Figure 1:**
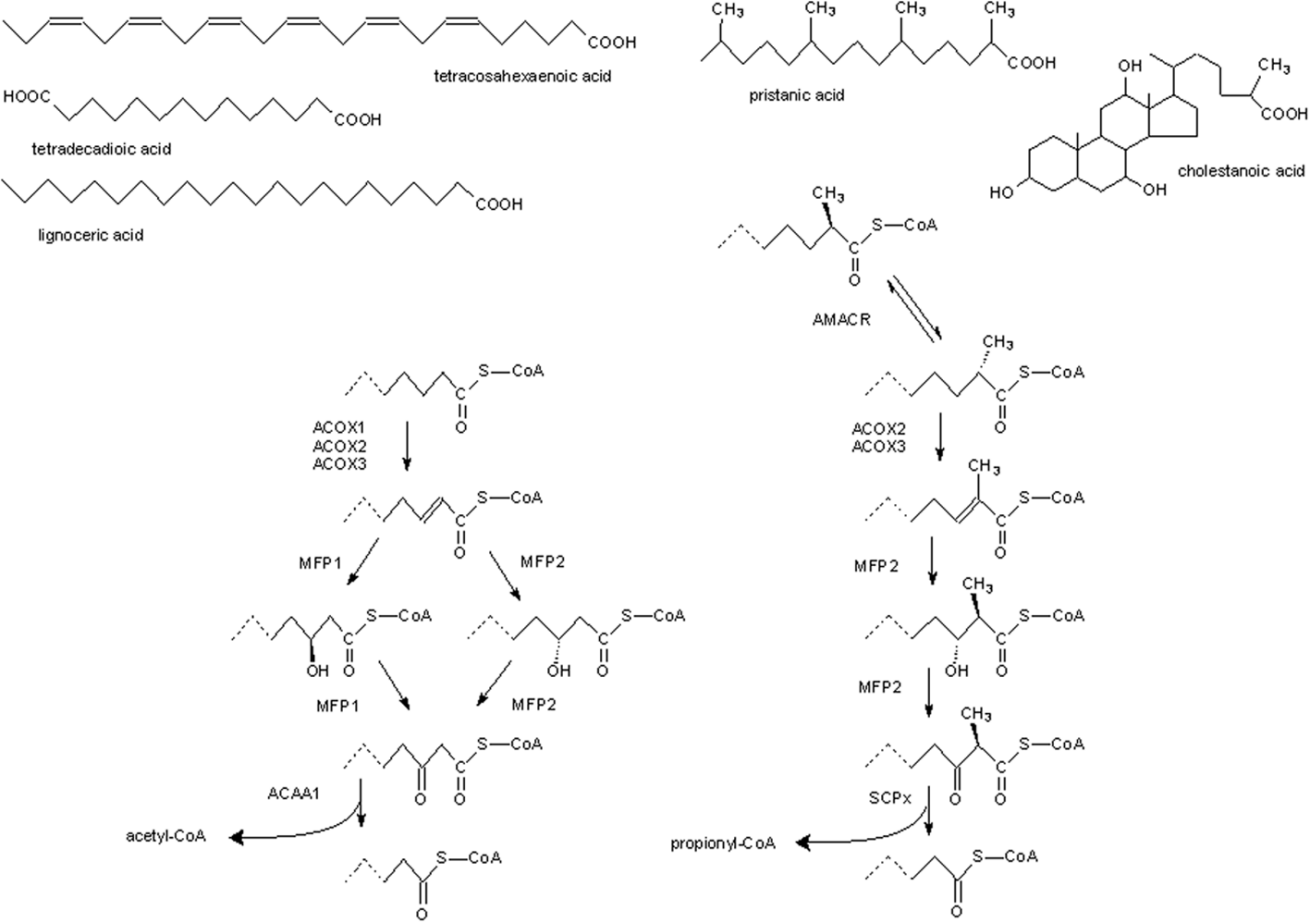
**Generalized scheme of peroxisomal β-oxidation in animals**. On **top**, structures of some fatty carboxylates that, after activation (not shown), are degraded by peroxisomal β-oxidation. At the **right**, enzymatic reactions/enzymes involved in degradation of substrates containing a 2-methylbranch, based on the situation in mammals. Most of these enzymes can act on straight chain substrates, shown at the **left**, as well. The latter compounds are also recognized by more selective enzymes which do not tolerate a 2-methylbranch. ACAA1, 3-ketoacyl-CoA thiolase; ACOX, acyl-CoA oxidase; AMACR, 2-methylacyl-CoA racemase; MFP, multifunctional protein; SCPx, sterol carrier protein X-thiolase.

Typically, peroxisomes can β-oxidize a broad range of natural, often also xenobiotic, compounds containing a fatty acyl side chain with or without a methyl-branch, in α-position of the carboxy-group. This process consists of a sequence of four reactions, resulting in shortening of the main chain of an acyl-CoA by 2 carbons (see Figure 1) (Van Veldhoven, 2010). In a first step, acyl-CoA is converted into 2-*trans*-enoyl-CoA by an acyl-CoA oxidase (ACOX), thereby producing H_2_O_2_. The number of ACOXs varies between species and ACOXs acting on 2-methyl-acyl-CoAs (ACOX2 and ACOX3 in mammals) are stereospecific, only the 2S-isoform is desaturated, hence an additional peroxisomal enzyme, 2-methylacyl-CoA racemase (AMACR), is required to convert the 2R-isoforms. The oxidation is followed by a hydration of the double bond by a 2-enoyl-CoA hydratase, a dehydrogenation by 3-hydroxyacyl-CoA dehydrogenase, and finally a thiolytic cleavage, generating acetyl-CoA (or propionyl-CoA in case of 2-methylbranched acyl-CoA) and a shortened acyl-CoA. Generally more than one enzyme can catalyze each of these steps, either homologous proteins as is the case for ACOXs or totally different proteins, e.g., thiolases encoded by the *Acaa1* or *Scp2* genes, or activities can reside in multi-enzymes (e.g., EHHAHD, also called multifunctional protein 1 (MFP1), HSD17B4, often called MFP2), which catalyze the hydration and dehydrogenation steps in a stereoselective manner. In mammals, a well-characterized β-oxidation pathway is the formation of C_24_-bile acids, starting from C_27_-bile acids (cholestanoic acids). In lower vertebrates, such as reptiles, some amphibia, and lungfishes, however, no C_24_-bile acids are found (Hofmann et al., 2010). On the other hand, the genomes of amphibia, bony fishes and various invertebrates like insects, bivalves, and sea urchins (but not nematodes), encode a peroxisomal AMACR, suggestive for a role of peroxisomes in breakdown of other isoprenoid derived carboxylates in these species.

**Figure 2:**
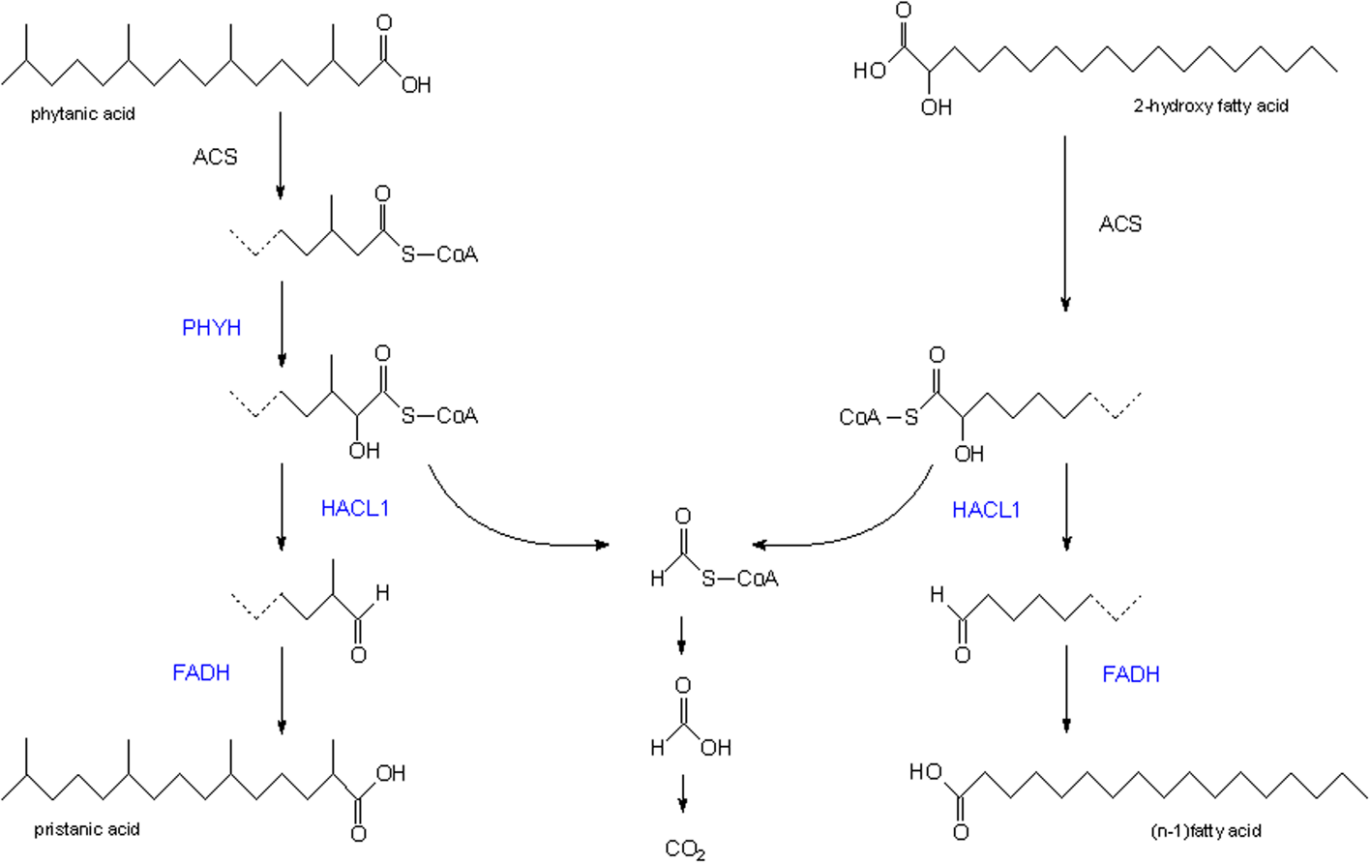
**Peroxisomal α-oxidation**. Scheme of the enzymatic reactions/enzymes involved in the peroxisomal breakdown of phytanic acid (**left**) and 2-hydroxy long chain fatty acids (**right**). Enzymes in blue are associated with peroxisomes. ACS, acyl-CoA synthetase; FADH, fatty aldehyde dehydrogenase; HACL1, 2-hydroxyacyl-CoA lyase; PHYH, phytanoyl-CoA α-hydroxylase.

α-Oxidation is a process whereby fatty acids are shortened by one carbon atom, amply documented for phytanic acid in man, a diet derived 3-methylbranched fatty acid, and less well-known for long chain 2-hydroxy fatty acids (Van Veldhoven, 2010) (see Figure 2). For phytanic acid, the process starts with the hydroxylation of phytanoyl-CoA at position 2 (by phytanoyl-CoA hydroxylase, PHYH), followed by a cleavage into formyl-CoA and pristanal, catalyzed by 2-hydroxyacyl-CoA lyase (HACL1). 2-Hydroxy long chain fatty acids do not depend on PHYH and are, after activation, shortened into a (n-1)fatty aldehyde by HACL1. This pathway is present in all mammals, and representative species of birds, reptiles, amphibian, fish, insects, nematodes, echinoderms, cnidaria, ascidia.

**Figure 3:**
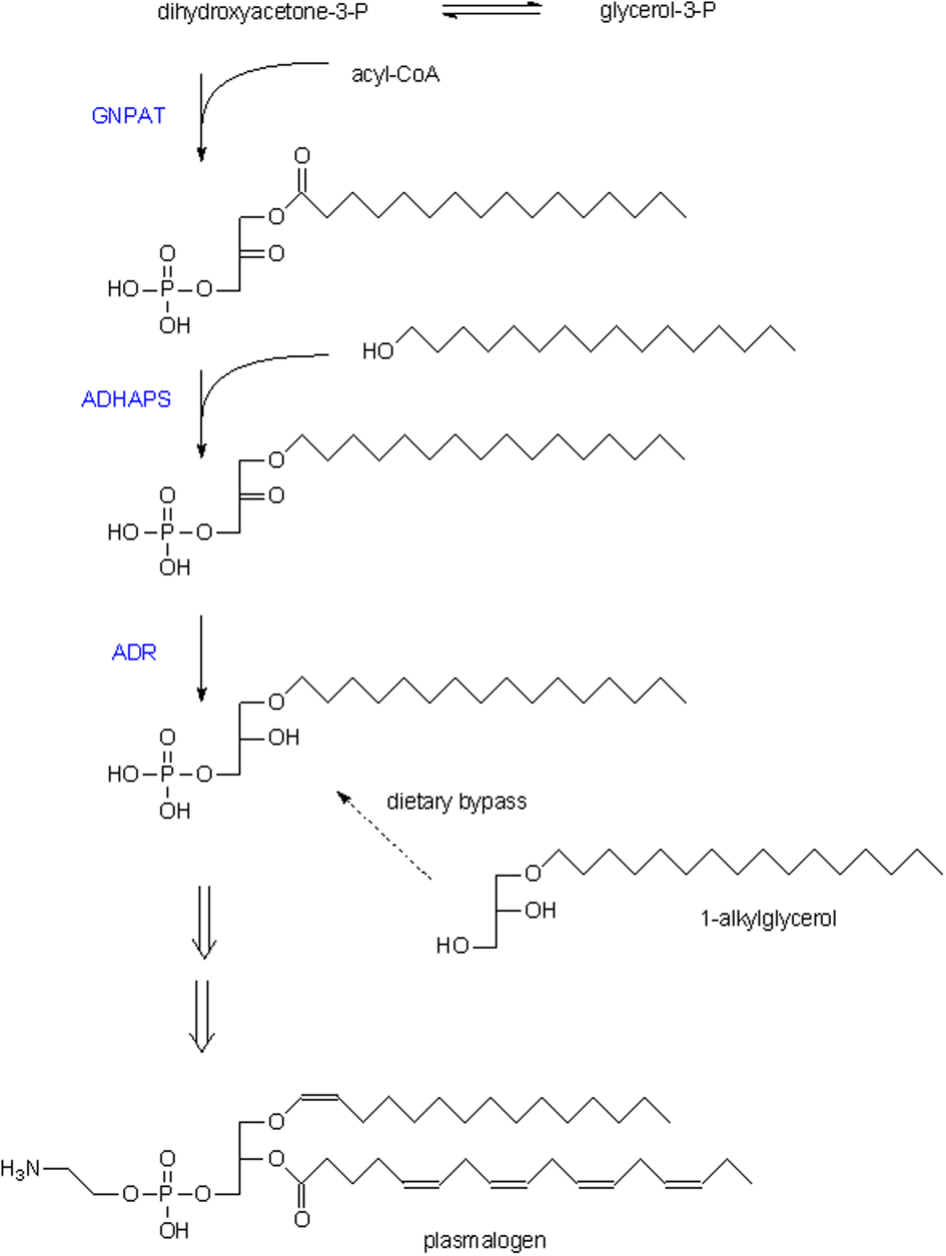
**Etherlipid biosynthesis**. Scheme of the reactions involved in formation of ether lipids, starting with dihydroxyacetone-phosphate. The long chain alcohol used by ADHAPS it generally 16–18 carbons, either saturated or with one double bond. Double arrows indicate multiple steps, catalyzed by ER-associated enzymes like headgroup addition and the introduction of the double bond in the 1-alkylchain. Generally, plasmalogens are enriched in PUFA at position 2 of the glycerol moiety (shown is 1-hexadecyl-2-arachidonoyl-plasmenylethanolamine). Dietary 1-alkylglycerol can bypass the peroxisomal steps in this pathway (dashed arrow). GNPAT, dihydroxyacetone-phosphate acyltransferase; ADHAPS, alkyl dihydroxyacetone-phosphate synthase; ADR, acyl/alkyl-dihydroxyacetone-phosphate reductase.

In contrast to the bulk of glycerolipids containing ester-linked fatty acids, a small portion of glycerolipids contains an ether bond, the precursor of which is formed by peroxisomal enzymes (see Figure 3). A first one, dihydroxyacetone-phosphate acyltransferase (GNPAT) generates an obligate precursor, 1-acyl-dihydroxyacetone-phosphate, a second one catalyzes the exchange of the acyl for an alcohol (alkyl dihydroxyacetone-phosphate synthase, ADHAPS). After reduction, the generated 1-alkylglycerol-3-phosphate follows the same anabolic routes as 1-acylglycerol-3-phosphate in the ER, leading to neutral and phosphoglycerolipids with a 1-alkyl group. In mammals, 1-alkyl-2-acylglycerophosphoethanolamine is desaturated just adjacent to the ether linkage, generating plasmenylethanolamine which can be converted to the choline analogue. Phospholipids with this vinylether group are better known as plasmalogens. Based on genomic information, the key enzymes GNPAT and ADHAPS are expressed in nematodes, cnidaria, echinoderms, insects, fish, amphibia, reptiles and birds. The presence of plasmalogens will however, depend on the expression of plasmanylethanolamine desaturase, an orphan enzyme not yet cloned. Besides mammals, plasmalogens have been identified in various animals including birds, amphibia, fish, insects, molluscs, marine worms, jelly fish, echinodermata, slime mold (Horrocks and Sharma, 1982).

Depending on species, peroxisomes or related organelles (glyoxysomes) are more or less actively involved in glyoxylate metabolism and in the degradation of purines (purine oxidation pathway or ureide pathway). Depending on the phylogenetic position of the species, purines are degraded till the level of ureum (amphibian, fish) or only till uric acid (man).

### Peroxisome biogenesis

**Figure 4:**
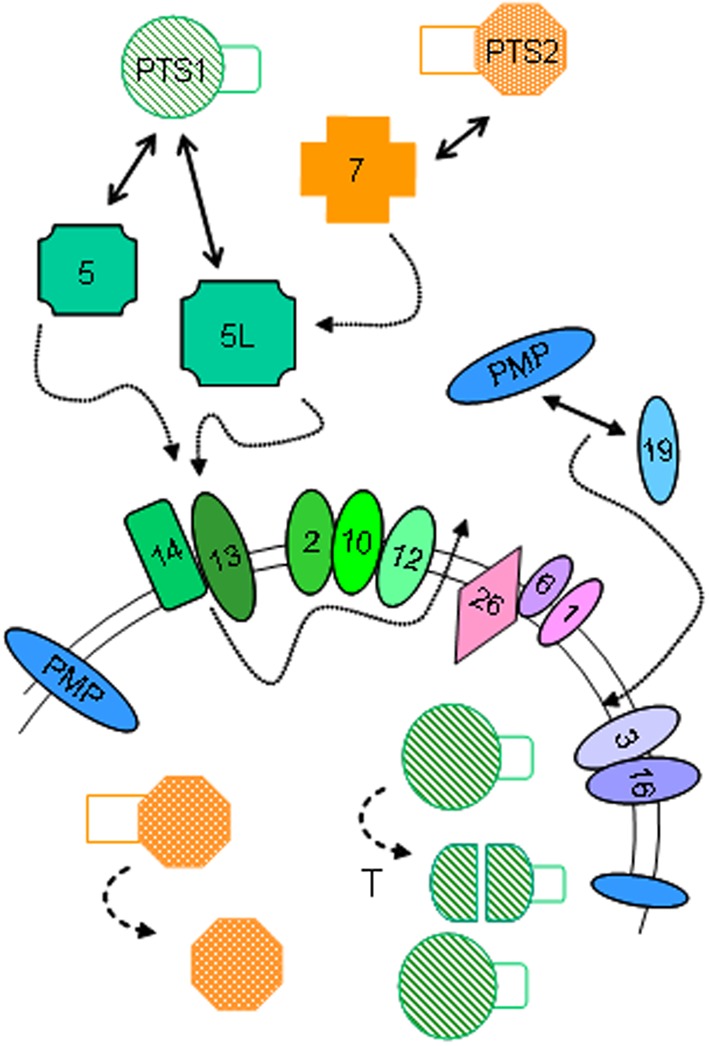
**Peroxisome biogenesis in animals**. Simplified scheme for the import of PTS1-proteins, PTS2-proteins and integral peroxisomal membrane proteins (PMP) in animals and the involved peroxins, indicated by their PEX number. Role of ubiquitination of PEX5 and farnesylation of PEX19 is not shown. After import, some PTS1-proteins are processed by TYSND1, a peroxisomal protease (T), while the signal peptide of PTS2-proteins is cleaved off. Adapted from Van Veldhoven (2010).

Proteins involved in the formation (biogenesis) of peroxisomes were first identified in yeast (Erdmann et al., 1989), and the major players in this process are rather well-conserved throughout the different kingdoms. In yeast and lower eukaryotes, however, more peroxins are found that are involved in fission/fusion processes and regulation of the number of peroxisomes^2^, which is related to the fact that these species must be able to adapt their intracellular organelles quickly to changes in their environment. Briefly for animals, peroxisomal matrix proteins, synthesized in the cytosol, are captured by binding partners that recognize a specific motif within their primary sequence, either a C-terminal tripeptide, better known as Peroxisome Targeting Signal 1 (PTS1) which is recognized by PEX5, or an N-terminal nonapeptide (PTS2), bound by PEX7 (see Figure 4). Upstream residues of PTS1, often referred to as SKL-sequence, influence binding to Pex5, hence a more in depth analysis of the interaction has broadened PTS1 to a dodecamer (Brocard and Hartig, 2006). In all species investigated, only a minority of matrix proteins contain PTS2, and in certain species, such as nematodes (*C. elegans;* Motley et al., 2000), diatoms (*Phaeodactylum tricornutum;* Gonzalez et al., 2011), and insects (*Drosophila;* Faust et al., 2012), PEX7 is even missing. In those organisms, the classical PTS2 proteins are still associated with peroxisomes, but are decorated with PTS1 (de Vet et al., 1998; Motley et al., 2000; Faust et al., 2012).

PEX5-PTS1 cargoes dock to the peroxisomal membrane via PEX14/PEX13, and are subsequently translocated through the bilayer. At the matrical side of the membrane, the cargo is released and PEX5 recycles to the membrane where it will undergo ubiquitination mediated by the RING-finger proteins PEX2, PEX10, and PEX12, and extracted back to the cytosol via PEX1/PEX6 in an ATP-dependent manner.

PEX7-PTS2 cargo also binds to PEX14, not directly but mediated via a long isoform of PEX5 (PEX5L) in mammals and other vertebrates (Figure 4). In fungi, the function of the latter is taken over by PEX18/PEX21 (Dodt et al., 2001).

Membrane biogenesis is depending on PEX19, PEX3, and (in animals) PEX16 (Fujiki et al., 2006). PEX19, a mainly cytosolic protein, plays a chaperone like function and binds most newly synthesized integral peroxisomal membrane proteins (PMP), and docks to PEX3, an integral peroxisomal membrane protein.

Finally, size and abundance of peroxisomes are regulated by PEX11 proteins, which play also a role in elongation of the organelles (Thoms and Erdmann, 2005; Koch et al., 2010). In mammals three isoforms are known, in lower animals only one (Table 1).

**Table 1:** **Overview of peroxins in animals used for peroxisomal research and in man**.

**Peroxin^2^**	**Description**	**Domain**		***Caenorhabditis elegans^1^***	***Drosophila melanogaster***	***Danio rerio***	***Mus musculus***	***Homo sapiens***
PEX1	AAA-ATPase		gi	25153574	21355121	283046720	61657895	4505725
			NP	NP_510386.2	NP_652016.1I	NP_001164306.1	NP_082053.1	NP_000457.1
			alias	prx-1 (isoform a); C11H1.4a	FBgn0013563	793906; ZDB-GENE-070530-1	ZWS1; 5430414H02Rik; E330005K07Rik	PBD1A; PBD1B; ZWS; ZWS1
			aa (MW)	996 (111997)	1006 (113739)	1237 (136665)	1244 (136613)	1283 (142737)
PEX2	E3 ligase	Zinc RING finger	gi	133931002	21355975	189536742	254028168	4506343
			NP	NP_502201.2	NP_648210.1	XP_684073.2	NP_001156773.1	NP_000309.1
			alias	prx-2; ZK809.7	CG7081; DmelPex2; Dmel; CG7081	ZDB-GENE-070530-2	D3Ertd138e; PAF-1; PMP35; Pxmp3	PAF1; PBD5A; PBD5B; PMP3; PMP35; PXMP3; RNF72; ZWS3
			aa (MW)	273 (31194)	281 (32239)	312 (35000)	305 (34813)	305 (34765)
PEX3	PEX9 docking factor		gi	193209553	21357431	41055494	9910484	4505727
			NP	NP_001123111.1	NP_648753.1	NP_956522.1	NP_064345.1	NP_003621
			alias	prx-3; C15H9.8	CG6859; DmelPex3; Dmel\CG6859; FBgn0036484	zgc:56313; fd60g05.y1; ZDB-GENE-040426-979	1700014F15Rik; 2810027F19Rik	PBD10A; TRG18
			aa (MW)	353 (39754)	385 (43562)	364 (41427)	372 (42093)	373 (42009)
PEX5	PTS1-receptor	TPR	gi	71983707	24639189	41055947	472339081	196259772
			NP	NP_001022019.1	NP_569949.3	NP_957450.1	NP_001264259.1	NP_001124496.1
			alias	prx-5; C34C6.6	CG14815; DmelPex5; Dmel\CG14815; EG:63B12.5	PXR1; zgc:56318; ZDB-GENE-040426-981	AW212715; ESTM1; PTS1R; Pxr1; X83306	PBD2A; PBD2B; PTS1-BP; PTS1R; PXR1
			aa (MW)	502 (55344)	559 (62994)	600 (67012)	602 (66675)	602 (66699)
PEX5L	PTS1-receptor	TPR	gi	absent	absent	absent	113930737	196259774
			NP				NP_033021.2	NP_001124497.1
			alias				AW212715; ESTM1; PTS1R; Pxr1; X83306	PBD2A; PBD2B; PTS1-BP; PTS1R; PXR1
			aa (MW)				639 (70625)	(639) 70734
PEX6	AAA-ATPase		gi	17562804	78707192	326678870	21703962	194018488
			NP	NP_504268.1	NP_001027403.1	XP_001332652.4	NP_663463.1	NP_000278.3
			alias	prx-6; CELE_F39G3.7	CG11919; CG30019; DmCG11919; DmelPex6; Dmel\CG11919	ZDB-GENE-081104-252	AI132582; mKIAA4177; PAF-2; D130055I09Rik; peroxisomal-type ATPase 1	PAF-2; PAF2; PBD4A; PDB4B; PXAAA1
			aa (MW)	720 (81223)	897 (100990)	865 (94312)	981 (104418)	980 (103930)
PEX7	PTS2-receptor	WD40	gi	absent	24661084^*^	61806636	6679283	4505731
			NP		NP_648251.1	NP_001013550.1	NP_032848.1	NP_000279.1
			alias		CG6486; DmelPex7; Dmel\CG6486	zgc:103552; ZDB-GENE-050320-105	MmPEX7	PBD9B; PTS2R; RCDP1; RD
			aa (MW)		339 (37486)	314 (34818)	318 (35371)	323 (35761)
PEX10		Zinc RING-finger	gi		392894943	54400490	109150414	24797089
			NP		NP_001021200.2	NP_001005994.1	NP_001035866.1	NP_722540.1
			alias		C34E10.4a^3^	zgc:103520	AV128229; Gm142	NALD; PBD6A; PBD6B; RNF69
			aa (MW)		314 (35871)	318 (37181)		346 (39083)
PEX11A	Peroxisome elongation Peroxisome constriction		gi	17506083	??	156739285	6755034	4505717
			NP	NP_493273.1		NP_001096590.1	NP_035198.1	NP_003838.1
			alias	prx-11; CELE_C47B2.8		si:dkeyp-84g1.1; 565760; ZDB-GENE-050419-121	PEX11alpha	hsPEX11p; PEX11-ALPHA; PMP28
			aa (MW)	214 (23780)		246 (27867)	246 (28022)	247 (28222)
PEX11B	Peroxisome elongation Peroxisome constriction		gi	??	19922346	113951761	241666483	4505719
			NP		NP_611071.1	NP_001039319.1	NP_001155859.1	NP_003837.1
			alias		CG8315; DmelPex11; Dmel\CG8315	zgc:153402; 566742; ZDB-GENE-060825-289	PEX11beta; Pex11pbeta	PEX11-BETA; PEX14B
			aa (MW)		241 (27007)	266 (29496)	259 (28579)	259 (28300)
PEX11G	Peroxisome elongation Peroxisome constriction		gi	??	28571837^4^	71834488	21735445	18087833
			NP		NP_651137.3	NP_001025343.1	NP_081227.1	NP_542393.1
			alias		BcDNA:RE30473; Dmel\CG13827	63203; ZDB-GENE-050913-79	1810022F11Rik; 1810049N02Rik; Pex11g; Pex11gamma	
			aa (MW)		233 (26208)	240 (26210)	241 (27021)	241 (26505)
PEX12		RING Zinc finger, C3HC4 type	gi	17551466	24580706	41055606	19527244	4505721
			NP	NP_509908.1	NP_608546.1	NP_956499.1	NP_598786.1	NP_000277.1
			alias	prx-12; F08B12.2	CG3639; DmelPex12; Dmel\CG3639	zgc:56182; 393174; ZDB-GENE-040426-929	AI451906	PAF-3; PBD3A
			aa (MW)	359 (41158)	297 (34413)	303 (33979)	359 (40502)	359 (40666)
PEX13	Docking PTS-cargo complex	SH3	gi	17533615	20129941	41055287	31543471	4505723
			NP	NP_495513.1	NP_610850.1	NP_956939.1	NP_076140.2	NP_002609.1
			alias	prx-13; F32A5.6	CG4663; Dmel\Pex13; Dmel\CG4663; FBgn0033812	zgc:66124; ZDB-GENE-040426-1544	2610008O20Rik	NALD; PBD11A; PBD11B; ZWS
			aa (MW)	330 (35635)	440 (46529)	416 (45338)	405 (44479)	403 (43999)
PEX14	Docking PTS-cargo complex		gi	17541806	21355205	292627105	9790153	4758896
			NP	NP_502097.1	NP_649253.1	XP_688421.4	NP_062755.1	NP_004556.1
			alias	prx-14; R07H5.1	CG4289; DmelPex14; Dmel\CG4289	559933; ZDB-GENE-060130-169	R75137	dJ734G22.2; NAPP2; PBD13A; Pex14p
			aa (MW)	258 (28025)	280 (30673)	422 (46234)	376 (41077)	377 (41106)
PEX16			gi	Absent	21355481	68448487	254750742	254750742
			NP		NP_649252.1	NP_001020340.1	NP_660104.2	NP_660104.2
			alias		CG3947; DmelPex16; Dmel\CG3947	im:6894523; zgc:112248		PBD8A; PBD8B
			aa (MW)		341 (39228)	335 (38424)	336 (38546)	336 (38546) (splice form; only on EST!)
PEX19	Cytosolic chaperone; PMP import receptor	CAAX-box	gi	17553610	24583827	62899043	226958490	4506339
			NP	NP_498947.1	NP_609547.2	NP_001017399.1	NP_075528.3	NP_002848.1
			alias	F54F2.8	BEST:GH03076; CG5325; DmelPex19; Dmel\CG5325	wu:fb40d12; wu:fc41h09; zgc:110675	Pxf	D1S2223E; HK33; PBD12A; PMP1; PMPI; PXF; PXMP1
			aa (MW)	282 (30857)	292 (31175)	288 (31412)	299 (32602)	299 (32676)
PEX20	Cytosolic chaperone		gi	??	386768875	Absent	Absent	Absent
			NP alias		NP_001245818.1^5^			
					CG3696; DmelPex20; Dmel\CG3696; EK2-4; kis; Su(Pc)21AB			
			aa (MW)		5343 (575803)			
PEX23	Peroxisome proliferation; peroxisomal growth regulation		gi	Absent	24667330	Absent	Absent	Absent
			NP		NP_730508.1^6^			
			alias		CG18565; CG32226; CG6468; DmelPex23; Dmel\CG32226			
			aa (MW)		1350 (149356)			
PEX26	Anchor for PEX1 and PEX6 to peroxisome membrane		gi	??	??	41053983	21311973	189083737
			NP	??	??	NP_956214.1	NP_083006.1	NP_001121121.1
			alias			fk41g06; wu:fk41g06; zgc:64014	4632428M11Rik; AI853212	PBD7A; PBD7B; PEX26M1T; Pex26pM1T; FLJ20695
			aa (MW)			313 (34257)	305 (33885)	305 (33767)

1 *It should be noted that in the Worm database, *prx-number* has been proposed as acronym for peroxisome assembly factors given confusion with *pex* (pachytene exit defect). However, various entries related to *prx* are linked to both PeroxidoRedoXins and PeRoXisome assembly factors, given use of similar acronym*.

2 *Peroxins, not present in animals, include PEX4, PEX8, PEX15, PEX17, PEX18, PEX21, PEX22, PEX25, PEX27, PEX28, PEX29, PEX30, PEX31, PEX32, PEX34 (all present in yeasts), PEX9, PEX20, PEX23 (*Yarrowia sp*.), PEX24 (yeasts, plants), PEX33 (*Neurospora sp*.)*.

3 *The *C34E10.4* locus produces a primary transcript coding for two different proteins, PEX10 (at the 5′; C34E10.4a) or WARS-2 (at the 3′; C34E10.4b)*.

4 *In addition to this entry, another PEX11 like protein (201 amino acids, MW 22671) is encoded by the fly genome. It concerns NP_995800.1 (gi 45552555), also named CG33474; Dmel\CG33474, which is most similar to PEX11G*.

5 *One of the six different isoforms encoded by *CG3696*, nowadays referred to as *kismet*; homologous to the human CHD7 (chromodomain-helicase-DNA-binding protein 7)*.

6 *Whether NP-730508.1 represents the counterpart of *Yarrowia* PEX23 or yeast PEX31 is questionable; they all contain a Dysferlin domain, but likely this entry is the counterpart of TECP1 (tectonin beta-propeller repeat-containing protein 1)*.

?? *not present in database, likely absent*.

* *homologue, but functionality not proven*.

## Models

In the following sections, laboratory animals in which peroxisome biogenesis has been studied will be described. In Table 1, gene symbols and alternative names for peroxins in these animals are listed. Given differences in life cycle and organogenesis, the development of these animals will be shortly described, and specific metabolic roles of peroxisomes, if documented, will be highlighted.

### Nematodes

A fertilized *Caenorhabditis elegans* egg develops into a small worm within the shell, in about 10–12 h. In the preceding 6 h (organogenesis/morphogenesis stage), the spheroid embryo started to elongate while its three germ layers differentiate into organs. After hatching, the post-embryonic development will start and the animal will pass, if food is present, through four larval stages (L1–L4, separated by 7–9 h) to reach sexual maturation, generally as a hermaphrodite, about 1.2 mm long, and will start to produce eggs. The cycle from egg to egg is therefore about 3 days; life span of the worm is 2 weeks.

In the adult nematode, peroxisomes are mainly present in the epithelial cells of the digestive tract, one of the largest organs, and in the pharyngeal gland (Yokota et al., 2002). In the gut, their volume density is 1.86/100 μm^2^ cytoplasm, similar to that in rat liver. Similar to rodents, fibrates increase the number of peroxisomes (Yokota et al., 2002). Based on the fluorescence pattern of animals expressing CFP-SKL, larvae contain more and larger peroxisomes than adult worms (Petriv et al., 2002).

In *C. elegans*, peroxisomal β-oxidation serves to generate acyl-CoAs used for the synthesis of dauer pheromone, also called daumone, a mixture of ascarosides which are excreted when the larvae are exposed to a hostile environment to block further development. Ascarosides are glycolipids and, in the case of dauer pheromone, consist of a hydroxylated medium chain fatty acid such as 6-hydroxyheptanoic acid or 8-hydroxy-2-nonenoic acid, O-glycosidically linked to ascarylose (3,6-didesoxymannose). Particularly daf-22 and dhs-28 (Butcher et al., 2009; Joo et al., 2009), the nematode counterparts of SCPX and the dehydrogenase moiety^3^ of D-specific MFP2, respectively, and acox-1 (Joo et al., 2010), one the seven nematodal ACOX proteins, are required for dauer pheromone production.

Regarding peroxisome biogenesis, it should be mentioned that the genome of *C. elegans* (and other nematodes) does not encode PEX7 (Motley et al., 2000). According to Thieringer et al. (2003) PEX16 is also missing and only one PEX11 isoform is present (Table 1).

During various large screenings by RNAi soaking, feeding or injection experiments, different peroxins were hit, however, the phenotype of the offspring was only minimally scored and the efficacy of silencing not investigated (see Table 2 and associated references). Moreover, these screens display some variability between approaches, are known to give rise to false negatives, and silencing is less effective in the nervous system. Efficacy can be increased by performing screens in the *rrf-3* mutant, a strain being hypersensitive to RNAi, likely due to longer half life of RNAi (Simmer et al., 2003). Overall these screens, certainly those by Simmer et al. (2003) and Sonnichsen et al. (2005), indicate that normal larval development depends on functional peroxisomes (Table 2).

**Table 2:** **Overview of large scale silencing screens in *C. elegans* affecting peroxins**.

		***Pex1***	***Pex2***	***Pex3***	***Pex5***	***Pex6***	***Pex10***	***Pex11***	***Pex12***	***Pex13***	***Pex14***	***Pex19***
**References**	**Method**											
Gonczy et al., 2000	Injection in gonads of dsRNA targeting genes of chromosome III						Slow growth					Larval arrest
Maeda et al., 2001	dsRNA soaking					Sick						
Kamath et al., 2003	Feeding *E. coli* expressing dsRNA	N	Slow growth	N	Slow growth; clear	N	Slow growth		Slow growth; clear	Slow growth	Slow growth	Slow growth
Simmer et al., 2003	Feeding *E. coli* expressing dsRNA in *rrf-3* strain	Slow growth	Slow growth	Slow growth	Larval arrest	Slow growth	Slow growth		Slow growth	Larval arrest	Larval arrest	Larval arrest
Ashrafi et al., 2003	Bacterial feeding of L1 larvae; analysis of fat content				Reduced fat content							
Rual et al., 2004	Feeding at the first larval stage (L1) *E. coli* expressing inducible hairpin RNAi	No		No	None	Long	No	No	Embryonic lethal	No	No	No
Sonnichsen et al., 2005	Injection dsRNA into young adult worms; examination phenotype of F1 progeny	No		Early larval arrest; defective early embryogenesis	Embryonic lethal	No	Early larval lethal	No	No	Embryonic lethal	Early larval arrest	Early larval lethal
Sieburth et al., 2005	Feeding bacteria expressing dsRNA to larvae; screening adults for decreased acetylcholine secretion (aldicarb resistance)		Aldicarb resistant									
Fernandez et al., 2005	Soaking of L4 stage larvae; with dsRNA corresponding to ovary expressed genes; progeny						Embryonic lethal	N			Embryonic lethal	Embryonic lethal
Curran and Ruvkun, 2007	Bacterial dsRNA feeding of L4-stage larvae (*eri-1(mg366)* strain); Screening life span of adult				Extended life span; Fat content reduced							
Byrne et al., 2007	Bacterial dsRNA feeding to L3-L4 stage worms; progeny and growth				Organism development variant							
Ceron et al., 2007	Bacterial dsRNA feeding to L1 larvae (*lin-35(n2239)* strain); progeny				Larval arrest; reduced brood size							

*N, no abnormalities reported; clear, animals appear unusually transparent when compared to control; long, animals are longer and thinner than control animals at the same developmental stage; sick, animals exhibit some combination of abnormal features relating to size, movement, body integrity, pigmentation, viability, fertility; larval arrest: development halted at any larval stage, failure to reach adulthood; slow growth, any variation that causes a reduction in growth rate compared to control*.

In more in depth investigations silencing dsRNAs were injected into the gonads of young adult hermaphrodites, followed by scoring of their effect on the progeny. Rachubinski and coworkers found that RNAi inactivation of *Pex5*, *Pex12*, *Pex13*, and *Pex19* greatly reduced the percentage of adult progeny, at 3 days following injection of dsRNA, most progeny being developmentally delayed and still at the L1, L2, or L3 larval stage (Petriv et al., 2002) Targeting of *Pex6*, *Pex1*, or *Pex2* was without effect, but the employed dsRNAs did also not affect the peroxisomal import of a fluorescent PTS1-protein (CFP-SKL). In contrast, injection of dsRNA targeting *Pex5*, *Pex13*, or *Pex19*, caused a cytosolic fluorescence of the reporter. Silencing of *Pex12* resulted in fewer but larger peroxisomes.

**Figure 5:**
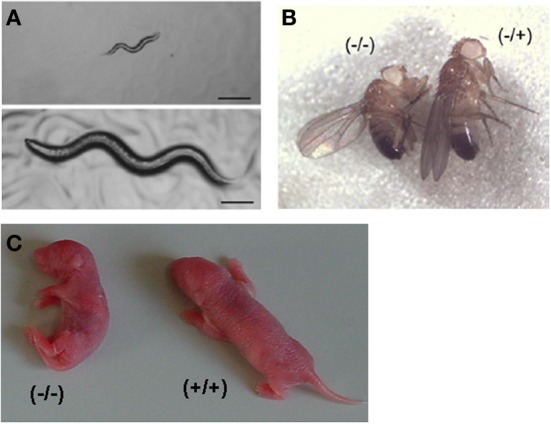
**Developmental delay in peroxisome deficient animals. (A)** Developmental arrest of *C. elegans* at the L1/L2 stage by *Pex5* RNAi (top panel), compared to wild type nematode (bottom panel), being photographed 3 days after being laid. Bar, 10 μm. Reproduced/adapted with permission from Thieringer et al. (2003). **(B)** Reduced body size and weight of an adult male homozygous *pex16^1^* fruitfly compared to heterozygous animal. Taken from Nakayama et al. (2011). **(C)** Appearance of newborn mice pups, showing severe hypotonia and growth delay in a *Pex5*^−/−^ pup compared to a wild type littermate.

Thieringer et al. (2003) reported similar experiments. Blocking either *Pex5*, *Pex6*, *Pex12*, *Pex13*, or *Pex19* caused an arrest of the growth of their progeny at the L1 larval stage (Figure 5A). The arrested worms were viable, and resumed growth after 2–8 days, likely depending on quantity and stability of injected DNA, and developed into normal worms.

Development seems less dependent on *Pex10*. During an ethyl methanesulfonate mutagenesis screen for genes affecting lipid droplets, Zhang et al. (2012) could classify surviving mutants having enlarged lipid droplets into four complementation groups, one group being linked to *Pex10*. In the mutant strain (prx-10(hj21)), PTS1 import was affected. Although not discussed in their paper, development and morphology of the worm appeared normal (based on pictures of 1 day adult). Given that the three other groups were linked to peroxisomal β-oxidation enzymes (*maoc-1*, *dhs-28*, *daf-22*, respectively, corresponding to an enoyl-CoA hydratase, 3-hydroxyacyl-CoA dehydrogenase and SCP2-containing thiolase), one could wonder why only one peroxin was hit in this screen or whether the others remained undetected due to lethality or slower development.

The mechanism underlying the developmental problems was not addressed, but might indeed be caused by peroxisomal metabolic inactivity. *Pex5*(RNAi) prevents initiation of post-embryonic cell divisions, and normal cell migrations including those of neuronal cells, are blocked (Thieringer et al., 2003). This phenotype resembles that of starved larvae, therefore division might require a peroxisomal metabolite. Furthermore, larval development of nematodes seems to be dependent on etherlipid synthesis. Eight days after injecting gonads of adults with dsRNA directed toward ADHAPS, their offspring was still in the larval stage, whereas those injected with non-specific dsRNA produced mature adults (Motley et al., 2000). Similar findings were reported by Petriv et al. (2002). Also β-oxidation might play a role in development. Upon silencing of Δ3,5-Δ2,4-dienoyl-CoA isomerase (encoded by *Y25C1A.13*), an enzyme required for degradation of polyunsaturated fatty acids, or silencing of the three ABC half-transporters (encoded by *T02D1.5*, *C44B7.9*, and *C44B7.8)*, implicated in peroxisomal membrane translocation of fatty acids/acyl-CoAs, a similar phenotype is seen: no adult offspring three days after injection (Petriv et al., 2002). It should be noted, however, that silencing of peroxisomal thiolases, either the classical ones (encoded by *T02G5.4* and *T02G5.8*) or SCP2-containing thiolase (encoded by *daf-22*), had no effect (Petriv et al., 2002). Also important to mention is that the *C. elegans* dienoyl-CoA isomerase, thought to be the counterpart of mammalian ECH1 (Petriv et al., 2002), which is targeted to both mitochondria and peroxisomes, does not have a PTS1. This complicates the interpretation of these silencing experiments.

Whereas a defective peroxisome biogenesis affects early larval development, silencing of *Pex* genes at a later stage seems beneficial. An extended life span, 22.7 days compared to 16.22, was seen upon silencing of *Pex5* in L4 larvae in the *eri-1(mg366)* strain, a strain more sensitive to RNAi (Curran and Ruvkun, 2007). Similarly, Zhou et al. reported a 17% increase for *Pex13* and 8% for *Pex5* (15% for PMX4, a peroxisomal membrane protein), when silenced in 1 day old adult (Zhou et al., 2012). It is suggested that the longer survival is related to reduced generation of reactive oxygen species (ROS) when peroxisomes are less or not functional (see Fransen et al., 2012). The amount of measurable ROS is lowered in the *Pex5*, *Pex11*, or *Pex13* silenced animals. Strangely, and in contrast to most other screens, silencing of these peroxins in L1 larvae had no effect, but controls on RNAi efficacy are missing.

Summarizing, RNAi based data suggest that peroxins play a critical role in nematode development, but are less important in the adult stage. A drawback of this technique is, however, the variability. In the near future, more solid data on the role of peroxins in nematodes are expected, given the increasing availability of deletion mutants (C.elegans mutation consortium. 2012): a *Pex5* mutant (tm4948) with a 439 bp deletion is sterile^4^; a *Pex1* mutant (tm0392) with a 681 bp deletion is classified as lethal or sterile^5^.

### Fruitfly

About 21–22 h after fertilization (hpf), *Drosophila* larvae will hatch from the eggs. One distinguishes 17 steps during this period, known as Bownes stage numbers. In stage 6 (180–195 min), gastrulation starts, whereas formation of the Malphigian tubes (counterpart of kidney in mammals) starts in stage 10. In the late stage 11, the stomatogastric nervous system develops. During the subsequent larval stages, three in total, most of the organs/structures of the adult fly will develop, starting from imaginal discs. At the end of the third larval stage (120 hpf), metamorphosis starts, divided in a prepupal period and a pupal period, in total 4 days. Finally, the flies emerge from the pupal case (eclosion). They start mating 12 h after emergence and will live for about a month.

Based on fatty acid analysis of certain *Pex* mutants, very long chain fatty acids (VLCFA) are degraded via peroxisomal β-oxidation in fruitflies (Chen et al., 2010). Related to purine/xanthine metabolism, it should be noted that the eye pigment formation is dependent on peroxisomes. The rosy-506 eye-color mutant lacks xanthine dehydrogenase/oxidase, which is targeted to peroxisomes (Beard and Holtzman, 1987).

The genome of *Drosophila* encodes at least 15 peroxins (Chen et al., 2010; Mast et al., 2011), being homologous to mammalian peroxins. Whether orthologous of the fungal *Pex20* and *Pex23* are expressed (Mast et al., 2011), is questionable (see comments to the related entries in Table 1).

Based on RNAi in *Drosophila* S2 cells expressing GFP-SKL, silencing of *Pex1*, *Pex5*, *Pex13*, *Pex16* results in import deficiency, silencing of *Pex2*, *Pex3*, *Pex6*, *Pex12*, *Pex14* in impaired import. Interfering with *Pex11* or *Pex19* affects peroxisome number (reduced) and size (larger), whereas RNAi of the putative *Pex20* or *Pex23* has an opposite effect, more peroxisomes of smaller size (Mast et al., 2011).

Although for most of these peroxins, mutants with transposon P1-insertions were present in the repositories (see Table 3), these were not studied in depth. According to Spradling *et al*. (Spradling et al., 1999), the *Pex2*^*f*018^ allele was lethal, but this was later shown to be due to a second mutation (Chen et al., 2010). More recently, a library of RNAi transgenes, expressing inverted repeats causing conditional gene inactivation, became available, covering 88% of the predicted protein coding genes (Dietzl et al., 2007). For all fly peroxins, transgenic lines are available (unpublished data), but as far as known, not evaluated.

**Table 3:** **Overview of classical peroxin alleles in *Drosophila melanogaster***.

**Gene**	**Allele**	**Mutagenesis method**	**References**
*Pex1*	*Pex1^1^*	X-ray	http://flybase.org/reports/FBal0031854.html
	*Pex1^s4868^*	P{lacW} insertion	http://flybase.org/reports/FBti0009969.html
	*Pex1^S084807^*	P{lacW} insertion (chromosome 3)	http://flybase.org/reports/FBal0095307.html
	*Pex1^02402^*	P{PZ} insertion	http://flybase.org/reports/FBal0031019.html
*Pex2*	*Pex2^f0189^*	PBac{WH} transposase	http://flybase.org/reports/FBal0161076.html
	*Pex2^f01075^*	PBac{WH} transposase	http://flybase.org/reports/FBal0222659.html
*Pex3*	*Pex3^EY1920^*	P{EPg} insertion	http://flybase.org/reports/FBal0215913.html
	*Pex3^c02356^*	PBac{PB} transposase	http://flybase.org/reports/FBal0222963.html
*Pex5*	*Pex5^JC02^*	P{PZ} insertion	http://flybase.org/reports/FBal0244572.html
*Pex6*	*Pex6^EY09695^*	P{EPgy2} insertion	http://flybase.org/reports/FBal0176366.html
	*Pex6^f03888^*	PBac{WH} transposase	http://flybase.org/reports/FBal0222729.html
*Pex7*		none	
*Pex10*	*Pex10^NP7003^*	P{GawB} insertion	http://flybase.org/reports/FBal0225637.html
	*Pex10^c03596^*	PBac{PB} transposase	http://flybase.org/reports/FBal0225639.html
	*Pex10^DP01222^*	P{Mae-UAS.6.11} insertion	http://flybase.org/reports/FBal0238882.html
	*Pex10^EY23523^*	P{EPgy2} insertion	http://flybase.org/reports/FBal0245128.html
	*Pex10^G5094^*	P{EP} insertion	http://flybase.org/reports/FBal0220877.html
	*Pex10^MI04076^*	Mi{MIC}insertion	http://flybase.org/reports/FBal0264496.html
	*Pex10^c03579^*	PBac{PB} transposase	http://flybase.org/reports/FBal0225638.html
*Pex11*	*Pex11^LA00967^*	P{Mae-UAS.6.11} insertion	http://flybase.org/reports/FBal0184822.html
	*Pex11^f03235^*	PBac{WH} transposase	http://flybase.org/reports/FBal0184821.html
*Pex12*	*Pex12^f01300^*	PBac{WH} transposase	http://flybase.org/reports/FBal0185737.html
	*Pex12^Δ 303^*	P{EPgy2} insertion	http://flybase.org/reports/FBal0242101.html
*Pex13*	*Pex13^e02054^*	PBac{RB} transposase	http://flybase.org/reports/FBal0185582.html
	*Pex13^KG04339^*	P{SUPor-P} insertion	http://flybase.org/reports/FBal0134334.html
	*Pex14^EY02900^*	P{EPgy2} insertion	http://flybase.org/reports/FBal0156977.html
*Pex14*	*Pex14^EY02900^*	P{EPgy2} insertion	http://flybase.org/reports/FBal0156977.html
*Pex16*	*Pex16^EY05323^*	P{EPgy2}	http://flybase.org/reports/FBal0161441.html
	*Pex16^GS14106^*	P{GSV6} insertion	http://flybase.org/reports/FBti0106543.html
*Pex19*	*Pex19^DP00474^*	P{Mae-UAS.6.11} insertion	http://flybase.org/reports/FBal0238836.html
	*Pex19^EY21383^*	P{EPgy2} insertion	http://flybase.org/reports/FBal0192628.html
*Pex23*		none (see also Table 1)	

Related to fly development, and as far as studied in detail, PEX1, PEX3, and PEX13 appear critical. P-element insertion in *Pex1* (*pex1*^*s*4868^) (Chen et al., 2010; Mast et al., 2011) or in *Pex13* (*pex13*^*KG*04339^) (Chen et al., 2010), X-ray mutagenized *Pex1^1^* (Mast et al., 2011) or a deletion in *Pex3*, generated by P-element imprecise excision of *pex3*^*CG*6859^ (Nakayama et al., 2011), are lethal^6^ at the larval stage when homozygous. Expression of a wild type PEX1 rescues the *pex1*^*s*4868^ or *pex1^1^* mutants to survive past the second larval instar (Mast et al., 2011). *Pex1* mutant larvae displayed a delay in development, little coordinated locomotion, poor feeding, and died at the L1–L2 stage (Mast et al., 2011). Some larvae even died a few hours after hatching, being unable to crawl out of the eggshells. In the peripheral and central nervous system various abnormalities were documented. These include malformation of the ventral nerve cord (lack of or underdeveloped commissures, breaks in longitudinal connectivities), reduced number of motor neurons, disorganization of glia cells, loss and hypoplasia of peripheral neurons, malformation of eye discs. In the malphigian tubules, structural abnormalities were noticed.

A dsRNA screen was conducted in preblastoderm embryos to detect genes that affect embryonic nervous system development. Although 50% of the *Drosophila* genes were covered, only one peroxin was hit, i.e., PEX19. Silencing of *Pex19* resulted in disruption of the ventral nerve cord, misrouting of axons and disorganization of dorsal clusters of cells in the peripheral nervous system in stage 15–16 embryos (Koizumi et al., 2007).

**Figure 6:**
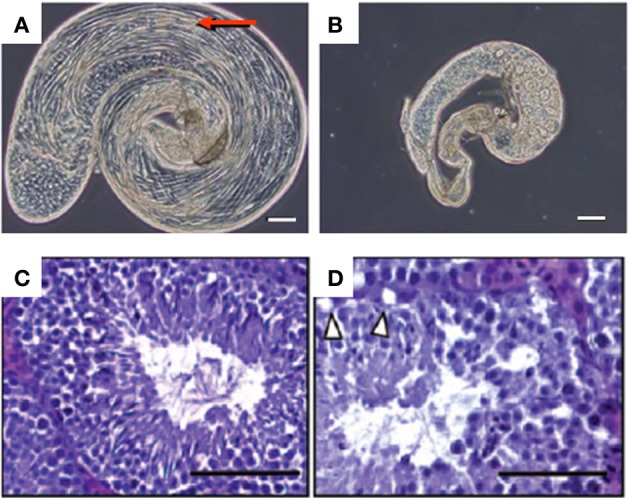
**Male fertility problems in peroxisome deficient animals. (A,B)** Phase-contrast micrographs of testes of fruitflies, with bundles of elongated spermatids (arrow) in a wild type **(A)** and arrest of germline cell maturation in a *pex16^1^* homozygous fruitfly **(B)**. Bar, 100 μm. Taken from Nakayama et al. (2011). **(C,D)** Hematoxylin-eosin staining of 7 weeks old testis of wild type **(C)** and Sertoli PEX5 knockout mice **(D)**, the latter showing lipid droplets that were emptied during the embedding procedure in the outer layer of the seminiferous epithelium (arrowheads) and reduced numbers of spermatozoa in the lumen of the tubuli. Bar 100 μm. Taken from Huyghe et al. (2006b).

Flies with insertional mutations in *Pex2(pex2*^*f*0189^ and *pex2*^*HP*35039^) (Chen et al., 2010), *Pex12* (*pex12*^*f*01300^) (Chen et al., 2010), *Pex1* (*pex1*^*S*4868^) (Zhou et al., 2012) or *Pex13* (*pex13*^*KG*04339^) (Zhou et al., 2012) or a deletion in *Pex10* (excision of P-element in *pex10*^*EY*23523^) (Chen et al., 2010) or *Pex16* (excision of *pex16*^*CG*3947^) (Nakayama et al., 2011) are viable. Fertility, however, was reduced in *Pex2*, *Pex10*, or *Pex12* female mutants and males were sterile (Chen et al., 2010). The latter phenotype was due to an arrest in the germ cell development at the level of the spermatocyte growth stage. Similarly, male fertility was compromised in the *Pex16* mutant (Nakayama et al., 2011). Testes of this mutant were smaller and did not contain mature sperm cells, although early spermatocyte cysts were still present, due to an arrest in the maturation of spermatocytes at the young apolar stage (Figures 6A,B). This arrest and the fertility could be rescued by overexpression of PEX16 in the cyst cells, although germ cells still lacked peroxisomes. Expression of PEX16 in the germline cells, however, did not rescue the spermatogenesis, indicating that peroxisomes in the somatic cysts cells play an important role in spermatogenesis (Nakayama et al., 2011). This is, however, in contrast to the *Pex2* mutant in which rescue of the germ cells normalized the phenotype (Chen et al., 2010). It is suggested that VLCFA, which show an age-dependent increase in *Pex10* mutants (2.9- and 3.9-fold for whole body C_26:0_ at 2 and 15 days, respectively), play a critical role in spermatogenesis in insects (Chen et al., 2010).

Although viable, the *Pex16* mutant adult flies were considerably smaller (30% females; 15% males) (Figure 5B) and their locomotion was affected, the latter likely being responsible for a severe reduction of their lifespan (to one third for females; to one-fourth for males) (Nakayama et al., 2011). Peroxisomes were still present in the malpighian tubule cells of *Pex16* mutant flies, but their number is greatly reduced. Not unexpectedly, the eye pigmentation was affected in *Pex16* mutants, resembling the *rosy* phenotype, and biochemically, an increase in (whole body) VLCFA levels was seen (2-fold for C_24:0_ in males, 3-fold in females). Histology of brain revealed a low density of dendritic trees in the lobula plate of the optic lobe; other cells in the optic lobe and other parts of the brain were unaffected. The dendritic reduction was already visible at the pupal stage and did not aggravate with age, suggesting a developmental problem, not a degeneration. Interestingly this defect can be rescued by expression of PEX16 in the fat body or in differentiated neurons (Nakayama et al., 2011).

The viability of the above mentioned *Pex1* and *Pex13* mutants can be explained by the nature of the mutation, affecting the promoter and resulting in lower expression (~20% of wild type) (Zhou et al., 2012). Interestingly, life span of these flies increased (16% in males; 13% in females), whereas their hydrogen peroxide levels were decreased. This is similar to findings reported in nematodes (see Nematodes). Along the same lines, *Pex19* expression was reported to be repressed (1.8-fold) when feeding flies 4-phenylbutyrate, a diet which extends their lifespan by 36% (Kang et al., 2002). The latter compound is known in the peroxisomal field by its ability to induce the expression of ABCD2 (Kemp et al., 1998), an ABC-transporter functionally related to ABCD1 which is mutated in X-ALD.

### Zebrafish

Given the translucency of the embryo and the short developmental period, zebrafish (*Danio rerio*) is an organism of choice for dynamic developmental studies. Gastrulation starts around 6 h post-fertilization (hpf), first somites are formed at 11 hpf, and at 24 hpf the embryo, surrounding the yolk sac, shows already the typical fish-like shape and tail and primary organs have been formed. In the subsequent day, the circulatory system and fins are formed. Cartilage development starts at 48 hpf, and at 3 days, fishes are self-supporting, first as larvae till 1 month of age, then juveniles till adulthood, around 90 days. Total life span is around 2 years.

Transcripts for peroxisomal matrix and membrane proteins can be detected starting at 24 hpf in the head region, whereas catalase-positive peroxisomes become visible in the liver and the pronephric duct in 4 days old fishes (Krysko et al., 2010). In adult fish, peroxisomes are most prominent in liver (Braunbeck et al., 1990; Krysko et al., 2010), renal proximal tubules (Krysko et al., 2010) and the intestinal epithelium (Krysko et al., 2010). For more information on expression in zebrafish during embryogenesis, the reader is referred to a large scale *in situ* hybridization screen (Thisse et al., 2004).

Similarly to rodents, zebrafish hepatic peroxisomes respond to peroxisome proliferators and an increased number is observed in liver when fishes are exposed to clofibrate (Venkatachalam et al., 2012) or phthalate esters (Ortiz-Zarragoitia et al., 2006).

Based on scattered information, the organelles are active in β-oxidation. Presence of ACOX1 was demonstrated (Ibabe et al., 2005; Morais et al., 2007) and the enzymes able to act on branched fatty acids, such as MFP2 (encoded by *hsd17b4*) and SCPX (encoded by *scp2a*), are expressed (Thisse et al., 2004), but apparently C_24_-bile acids are not formed in zebrafish, in contrast to other teleost fish (Hofmann et al., 2010). Based on genomic information, fish peroxisomes can synthesize etherlipids and contain an α-oxidation pathway.

Regarding peroxisome biogenesis, all classical peroxins are expressed in *D. rerio* (see Table 1), and based on high throughput analysis, *Pex3*, *Pex5*, *Pex7*, *Pex10*, *Pex14*, *Pex19* are ubiquitously expressed from 24 hpf on, with higher expression in the head region (Thisse et al., 2004).

Despite the wide spread use of morpholinos to interfere with expression in zebrafish, in only few reports, as far as documented, this technique was applied to peroxisome biogenesis. Injection of morpholinos, intended to block the splice sites in *Pex3* or *Pex13*, into one-cell embryos did not affect peroxisomal import. Subsequent RNA analysis revealed that these morphilinos did not eliminate exons, instead produced a short in frame insertion (*Pex3*) or deletion (*Pex13*) (Krysko et al., 2010). Blocking of the translation of *Pex13* was more effective to reduce the number of hepatic peroxisomes, but high doses were needed and not all of the injected embryos showed such response. A *Pex5* blocking morpholino had no effect at low dose, and caused embryonal death at higher dose. Finally, overexpression via mRNA injection of an N-terminal domain of (human) PEX3, having a dominant negative effect in human fibroblasts (Soukupova et al., 1999), did not affect biogenesis (Krysko et al., 2010). Coutinho et al. (2004) did not observe any abnormalities at 32 hpf when one cell stage embryos were injected with morpholinos directed against the 5′-end of *Pex19* (notochord differentiation or pigmentation were normal), the efficacy of the morpholino was, however, not controlled.

Although technically easy, the dilution of morpholinos or mRNA upon subsequent cell divisions, combined with the turnover of peroxisomes, half life estimated at 2 days in cultured mammalian cells (Huybrechts et al., 2009), is a major obstacle in the embryonic injection approach. In the near future, more solid data might emerge from analysis of insertional zebrafish mutants. Although tools to carry out large scale insertional mutagenesis and positional cloning in zebrafish were developed several years ago using mouse retroviral vectors (Gaiano et al., 1996; Golling et al., 2002) the number of created, annotated and available mutants, however, remains low. For a more targeted approach, engineered Zn-finger nucleases are a promising tool to create zebrafish knockouts (Foley et al., 2009).

### Mice

The intra-uterine development of mice takes 20–21 days. During this period, embryos are depending on the maternal circulation with regards to most nutrients. Examples of exceptions are brain poly-unsaturated fatty acids (PUFA) that are partly dependent on local synthesis (Janssen et al., 2000). At birth, organogenesis of most organs has been completed, except formation of the cerebellum which extends into the postnatal period and maturation of gonads before adulthood. After birth, pups are nursed and milk-fed till weaning, about 3 weeks later. At 6 weeks (females) or eight (males) of age, animals become sexually active and start to breed. Lifespan, under laboratory conditions, is 18–30 months.

In mammals, peroxisomal β-oxidation serves to generate PUFA and C_24_ bile acids. The first are implicated in many brain processes such as learning, memory, behavior; the latter are required for efficient uptake of lipophilic nutrients in the intestines. This pathway also shortens VLCFA, pristanic acid and dicarboxylic fatty acids (Van Veldhoven, 2010). Removal of the toxic phytanic acid requires an active α-oxidation. Plasmalogen deficiency in mammals is linked to a specific bone developmental problem, in man known as rhizomelic chondrodysplasia punctata (RCDP), and RCDP type I is linked to PEX7 deficiency.

Currently, the following peroxins have been inactivated in mice: PEX5 (Baes et al., 1997), PEX2 (Faust and Hatten, 1997), PEX11A (Li et al., 2002a; Weng et al., 2013), PEX11B (Li et al., 2002b), PEX13 (Maxwell et al., 2003), and PEX7 (Brites et al., 2003; Braverman et al., 2010). Mice lacking both PEX11A and PEX11B were also created (Li et al., 2002b), or lacking a peroxin together with another peroxisomal protein such as *Pex7^−/−^:Abcd1^−/−^* mice (Brites et al., 2009).

Given obvious similarities, PEX5, PEX2, and PEX13 deficiencies can be treated together, separately from the PEX7 knockout model. Considering that PEX11 proteins are not involved in peroxisome biogenesis *per se* and that this process is not affected in the *Pex11a^−/−^* and *Pex11b^−/−^* mice, but mainly their elongation and abundance, these models will not be discussed further in this chapter. Below we will summarize the main findings in the other mouse models [see also recent reviews by Baes and Van Veldhoven (2006, 2012)].

Related to PEX5, PEX2, and PEX13 deficient models, knockouts pups are born alive in the expected Mendelian ratio and without major deformities or skeletal malformations, suggesting a normal intra-uterine development (Baes et al., 1997; Maxwell et al., 2003). However, in case of PEX2 deficiency in an inbred 129 background, embryonic lethality was reported and only 20% of the pups are born (Faust and Hatten, 1997). In these three models, newborn pups are, however, growth retarded and severely hypotonic (Figure 5C), hence they do not feed and die 6–24 h after birth. Some *Pex2^−/−^* pups (20–30%), in a mixed Swiss Webster × 129SvEv background, survive for about 1–2 weeks (Faust and Hatten, 1997) and the postnatal survival can be improved by oral bile acid therapy (9% alive after 30 days) (Keane et al., 2007). The reason for this strain-dependent differences, although often seen in other mouse models, is not clear.

At closer inspection, there are some developmental problems, especially in the brain. Lamination of the cerebral cortex is affected due abnormal and delayed neuron migration (Baes et al., 1997; Faust and Hatten, 1997; Gressens et al., 2000). In the longer surviving *Pex2^−/−^* pups, dendritic arborization of the Purkinje cells in the cerebellum is reduced and their axons are dystrophic (Faust, 2003). Similar findings were seen in a *Pex5* and *Pex13* brain knockout (see further).

Finally, at the subcellular level, mitochondrial abnormalities were documented in liver (Baumgart et al., 2001; Keane et al., 2007) and lamellar lipid deposits were evident in the adrenocortical cells (Faust and Hatten, 1997).

Biochemically, various peroxisome dependent parameters are abnormal in pups with these *Pex* gene inactivations [accumulation of VLCFA, lack of plasmalogen, abnormal bile acids, shortage of docosahexaenoic acid (DHA)]. Changes in brain PUFA composition have been proposed to modify α-synuclein (Yakunin et al., 2010), which could contribute to the neuropathology. In whole brain extracts of these three models, Yakunin et al. (2010) showed increasing oligomerization and phosphorylation of α-synuclein. Such changes trigger intraneuronal deposition of α-synuclein (Lewy bodies), being a hallmark of synucleopathies such as Parkinson disease.

A different phenotype is seen in PEX7 deficient mice (Brites et al., 2003). Embryonic lethality is not seen, but these pups are also hypotonic and growth impaired (15–30% lower body weight at birth), and the majority (70%) dies before weaning (50% after 1 day, likely due to the hypotonia). The surviving animals do live till adulthood and longer, but males are infertile, the seminiferous epithelium being devoid of spermatogonia and spermatocytes. In brain, a delay in neuronal migration is seen, and ossification of distal bone elements of the limbs, skull and vertebrae, is defective. The amount of white, but not brown, adipose tissue is reduced (Brites et al., 2011). Bilateral cataracts develop 2 weeks after birth (Brites et al., 2011), the time pups open their eye lids. Biochemically, plasmalogens are depleted, phytanic acid cannot be degraded, and VLCFA oxidation is impaired in fibroblasts, but increased VLCFA levels are only found in spleen, spinal cord and neonatal brain (Brites et al., 2009).

In *Pex7* hypomorphic mice, in which *Pex7* transcripts are reduced to 5%, lifespan is normal (Braverman et al., 2010). The mice are still smaller, but are fertile. Their tissue content of plasmalogens is low but not absent, DHA in RBC is lowered and phytanic acid accumulates. Pathological findings include endochondral ossification defects, abnormalities in lens fibers and eye cataract (Braverman et al., 2010).

Feeding 6-weeks old *Pex7^−/−^* mice with 1-*O*-octadecylglycerol, an etherlipid which is bypassing the peroxisomal biosynthetic steps (see Figure 3), reveals that several phenotypic abnormalities are related to plasmalogen deficiency. The diet restores plasmalogen levels in non-nervous tissues. In parallel, testicular pathology is ameliorated (spermatogenesis was restored, although mature spermatozoa were still not detectable), and adipocytes displayed a normal size and fat content. When giving 1-*O*-octadecylglycerol to newborn pups, via supplementing it to the diet of the mother, testicular degeneration was prevented and cataract formation was absent or only unilateral and reduced to a small nuclear cataract (Brites et al., 2011). In the hypomorphic *Pex7^−/−^* mice, such treatment did not affect the cataracts (Braverman et al., 2010).

Severe bone abnormalities, a major hallmark in patients with PEX7 deficiency as reflected in their name (RCDP), are not observed in mice. Upon closer investigation, a delay, however, in endochondral bone formation was reported in both complete (Brites et al., 2003) and hypomorphic PEX7 (Braverman et al., 2010) deficient mice, likely due to a delayed maturation of chondrocytes at the pre-hypertrophic state, but further mechanistic insights were not generated.

Given the lethality of peroxin knockouts, especially of those with affected PTS1-import, developmental and behavioral studies are limited. This can be circumvented by conditional knock-out whereby peroxisomes are removed in specific tissues and/or at a certain stage. Tissue-specific removal of peroxisomes can be established by crossing mice containing a floxed *Pex* gene (Baes et al., 2002) with mice expressing cre in a promoter-specific manner. The promoter also determines the time point from when on the *Pex* gene is irreversibly inactivated in the targeted cells and their descendents. This technology was applied for *Pex5* creating mice lacking peroxisomes in the central nervous system (CNS) [*nestin-Cre*, in neural precursors from embryonic (E) day 11 (Hulshagen et al., 2008)], hepatocytes [alfafoetoprotein-*Cre*, from E10 (Krysko et al., 2007) and albumin-*Cre*, from birth (Peeters et al., 2011)]. By using a similar approach, brain specific PEX13 knockouts were obtained *(nestin-Cre*) (Müller et al., 2011).

*Pex5* was further inactivated in specific cell types by using appropriate Cre-expressing mice: Sertoli cells (*Amh-Cre*, from E14) (Huyghe et al., 2006b), oligodendrocytes (*Cnp-Cre*, from E14) (Kassmann et al., 2007), principal neurons in the forebrain (*Nex-Cre*, from E12) (Bottelbergs et al., 2010), and astrocytes (*Gfap-Cre* from E13) (Bottelbergs et al., 2010). The specific inactivation of PEX5 in adipocytes failed due to the non-selectivity of the *aP2* promoter driving Cre expression (Martens et al., 2012).

Overall, these studies indicate that absence of peroxisomes in adipose (Martens et al., 2012), neurons (Bottelbergs et al., 2010), astrocytes (Bottelbergs et al., 2010), or Sertoli cells (Huyghe et al., 2006b) does not compromise life span. Postnatal thriving, however, requires functional liver and brain peroxisomes. Moreover, absence in liver results in life threatening development of hepatocarcinomas (Dirkx et al., 2005), absence in brain shortens life span considerably to 6 months with 20% dead before 3 weeks for *Pex5-loxP:nestin-cre* (Hulshagen et al., 2008) or 35 days for *Pex13-loxP:nestin-cre* (Müller et al., 2011) mice. Of the different models with specific brain cell inactivation, the oligodendrocyte knockout represents the worst outcome: almost none of the affected animals survive 1 year of age (Kassmann et al., 2007). Its phenotype resembles that of a total deficiency of peroxisomes in the brain, but with delayed onset of demyelination, axonal loss and neuroinflammation. The latter encompasses a strong activation of the innate immune system with microglia reactivity and increased expression of pro-inflammatory markers (Kassmann et al., 2007; Bottelbergs et al., 2012). The biochemical factor(s) contributing to or causing this phenotype remain unclear. To which extent peroxisomal metabolites can be transferred from one cell type to another in brain, or from the body to the brain, is not fully established, but an important role of peroxisomes in neurons or astrocytes in pre- and postnatal life can be excluded.

For more information about these models, and how peroxin deficiencies affect brain, liver and testis, we refer to recent reviews (Baes and Van Veldhoven, 2006, 2012; Baes and Aubourg, 2009). It should be stressed that part of the pathology seen in these mouse models might be related to the, not yet completely understood, interplay between peroxisomes, their metabolites and other organelles. As initially observed in PEX5 (Baumgart et al., 2001) and PEX2 knockouts (Keane et al., 2007), and further documented in the *albumin-Cre*/*Pex5-loxP* mice (Dirkx et al., 2005), absence of peroxisomes in hepatocytes affects their mitochondria severely. Structural alterations are seen in the inner mitochondrial membrane, and its potential is collapsed. Activities of complex I, III, and V are reduced. In addition, lipid droplets and ER stress are noticed. Based on the upregulation of ATF3, ATF4, ATF6, and CHOP, the unfolded protein response pathway is activated in absence of peroxisomes (Dirkx et al., 2005). Similar findings were seen in liver of surviving PEX2 pups, the integrated stress response mediated by PERK and ATF4 signaling being activated (Kovacs et al., 2009). It is postulated that perturbed peroxisomal β-oxidation metabolites (e.g., bile acid (intermediates), dicarboxylic acids), are causative factors given the fact that ER stress is also seen in mice with β-oxidation defects (Huang et al., 2011).

## Conclusion

Although peroxisomes are not essential for cell functioning and survival, at the multicellular level they are indispensable as demonstrated by the different animal models treated in this chapter.

A common feature in animals with peroxisome biogenesis defects is a developmental delay, smaller size at hatching/birth and limited to very short lifespan (Figure 5). The reason for the delay is not clear. In most models, organogenesis seems to proceed normal, but the central nervous system appears sensitive to absence of peroxisomes (abnormal cerebellar lamination and delayed neuron migration in mice; malformation of the ventral nerve cord in fruitfly; block of neuron cell migration in L1 stage in nematodes). In a later stage of life, neuronal problems are manifested in reduced locomotion (larvae of insects) or coordination and motor skills (mice). In nematodes, normal larval development is dependent on ether lipids. In mice (and man), plasmalogen deficiency is compatible with prenatal development but the newborns exhibit already several abnormalities.

With regard to the nervous system, an intriguing question is to which extent myelinization/demyelinization and axonal integrity are linked to peroxisomes. Myelin, formed by the oligodendrocytes, is indeed enriched in metabolites related to peroxisomes (plasmalogens, VLCFA). It is therefore surprising that myelination is initially normal when peroxisomes are ablated from oligodendroglia and that in adulthood myelin becomes destabilized. Importantly, as both in the total brain and the oligodendrocyte knockout, degenerated axons are observed surrounded with a normal myelin sheet, it was postulated that oligodendroglial peroxisomes serve to support axons independent of myelination. This is further endorsed by the finding that peroxisomes are abundant in paranodes (Kassmann et al., 2011), sites where glia and axons interact. In this context, one should recall that in species in which axons are not myelinated such as fruitfly, neuronal abnormalities are seen when peroxisomes are ablated (Nakayama et al., 2011).

Another remarkable finding, although not studied in all models, is the male sterility, documented at least in *Drosophila* and in mice knockouts (Figure 6). In fly, peroxisomes of the cysts cell appear to be important for spermatogenesis (Nakayama et al., 2011), which is mirrored in mice where peroxisomes are necessary in the Sertoli cells (Huyghe et al., 2006b). In insects, the infertility was linked to accumulation of VLCFA, in mice experimental evidence points toward both an accumulation of VLCFA and VLCFA-PUFA (Huyghe et al., 2006b). The importance of normal peroxisomal β-oxidation for male fertility was further confirmed in ACOX1 (Fan et al., 1996) and MFP2 knockout mice (Huyghe et al., 2006b). In addition, a depletion of ether lipids also causes male infertility in PEX7 (Brites et al., 2011) and GNPAT (Rodemer et al., 2003) knockout mice.

Finally, related to aging and neurodegenerative diseases, and the emerging role of peroxisomes in ROS signaling (Titorenko and Terlecky, 2011; Fransen et al., 2012) scattered information derived from the animal models discussed above, suggest that less active peroxisomes in adulthood could positively contribute to longevity. This seems, however, in conflict with the general concept that the metabolic activity of these organelles becomes compromised during aging. On the other hand, it would be consistent with studies on the importance of catalase in aging. Improving the removal of peroxide in peroxisomes, by expressing an engineered catalase with a higher affinity for PEX5, delays the appearance of senescence markers in human fibroblasts (Koepke et al., 2007). Hence, not the peroxisomal metabolic activity, but the ratio of ROS-generation/removal (Fransen et al., 2013), might be a determining factor in aging.

### Conflict of interest statement

The authors declare that the research was conducted in the absence of any commercial or financial relationships that could be construed as a potential conflict of interest.

## Footnotes

^1^For the sake of consistency, the nomenclature guidelines as formulated for rodents (http://www.informatics.jax.org/mgihome/nomen/gene.shtml) are followed throughout this text, gene symbols being italicized, first letter capitalized, whereas the protein is referred to by the corresponding gene symbol in standard capitalized font. Guidelines related to nomenclature in other species can be found at following URLs: nematodes (http://www.wormbase.org/about/userguide/nomenclature#fda31g748bjh9c650ie2—10); fruitflies (http://flybase.org/static_pages/docs/nomenclature/nomenclature3.html#1.2.3.); zebrafish (https://wiki.zfin.org/display/general/ZFIN+Zebrafish+Nomenclature+Guidelines#ZFINZebrafishNomenclatureGuidelines-1.1); man (http://www.genenames.org).

^2^In *sensu stricto* of the original definition (Distel et al., 1996), these proteins should not be called peroxins.

^3^In contrast to most other higher eukaryotes, the two catalytic domains of MFP2 are expressed as separate proteins in *C. elegans* (Huyghe et al., 2006a).

^4^http://www.shigen.nig.ac.jp/c.elegans/mutants/DetailsSearch?lang=english&seq=4948.

^5^http://www.shigen.nig.ac.jp/c.elegans/mutants/DetailsSearch?lang=english&seq=392.

^6^The *pex1*^*S*084807^ and *Pex1*^02402^ alleles were reported to be semi-lethal and in homozygous third instar larvae necrosis was observed in salivary gland cells (Burmester et al., 2000).
